# Posttranscriptional Regulation of Gene Expression Participates in the Myelin Restoration in Mouse Models of Multiple Sclerosis: Antisense Modulation of HuR and HuD ELAV RNA Binding Protein

**DOI:** 10.1007/s12035-023-03236-8

**Published:** 2023-01-25

**Authors:** Vittoria Borgonetti, Nicoletta Galeotti

**Affiliations:** Department of Neuroscience, Psychology, Drug Research and Child Health (NEUROFARBA), Section of Pharmacology, University of Florence, Viale G. Pieraccini 6, I–50139 Florence, Italy

**Keywords:** HuR, HuD, ELAV RNA binding protein, Neuropathic pain, Myelin, Microglia, Experimental autoimmune encephalomyelitis (EAE)

## Abstract

Neuropathic pain is the most difficult-to-treat pain syndrome in multiple sclerosis. Evidence relates neuropathic pain to demyelination, which often originates from unresolved neuroinflammation or altered immune response. Posttranscriptional regulation of gene expression might play a fundamental role in the regulation of these processes. The ELAV RNA-binding proteins HuR and HuD are involved in the promotion of inflammatory phenomena and in neuronal development and maintenance, respectively. Thus, the aim of this study was to investigate the role of HuR and HuD in demyelination-associated neuropathic pain in the mouse experimental autoimmune encephalomyelitis (EAE) model. HuR resulted overexpressed in the spinal cord of MOG_35-55_–EAE and PLP_139-151_–EAE mice and was detected in CD11b + cells. Conversely, HuD was largely downregulated in the MOG–EAE spinal cord, along with GAP43 and neurofilament H, while in PLP-EAE mice, HuD and neuronal markers remained unaltered. Intranasal antisense oligonucleotide (ASO) delivery to knockdown HuR, increased myelin basic protein expression, and Luxol Fast Blue staining in both EAE models, an indication of increased myelin content. These effects temporally coincided with attenuation of pain hypersensitivity. Anti-HuR ASO increased the expression of HuD in GAP43-expressing cells and promoted a HuD-mediated neuroprotective activity in MOG–EAE mice, while in PLP–EAE mice, HuR silencing dampened pro-inflammatory responses mediated by spinal microglia activation. In conclusion, anti-HuR ASO showed myelin protection at analgesic doses with multitarget mechanisms, and it deserves further consideration as an innovative agent to counteract demyelination in neuropathic pain states.

**Graphical Abstract:**

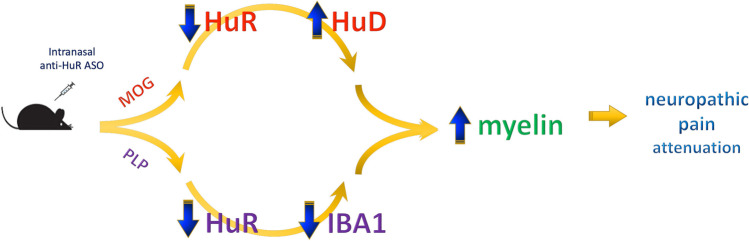

**Supplementary Information:**

The online version contains supplementary material available at 10.1007/s12035-023-03236-8.

## Introduction 

Multiple sclerosis (MS) is a chronic inflammatory condition of the central nervous system (CNS) characterized by demyelination with concomitant axonal and neuronal degeneration [1]. Among the symptoms and signs of MS patients, pain affects about 70% of patients, and neuropathic pain represents the most difficult-to-treat chronic pain syndrome [2]. Complete and effective relief from neuropathic pain is usually not achieved, and available treatments produce limited efficacy in a small portion (20–40%) of patients. In fact, therapy is often unsatisfactory due to poor efficacy and the appearance of unwanted side effects [3, 4]. Treatments for neuropathic pain do not focus on resolving the cause of the pain but rather on merely suppressing symptoms. Therefore, understanding the causal processes of neuropathic pain and their modulation could constitute an effective therapeutic strategy.

Nerve injury induces a pro-inflammatory response in the spinal cord in rodents and humans and can cause neuropathic pain [5, 6]. Spinal microglia have traditionally been implicated in this pro-inflammatory response and in the promotion of pain hypersensitivity [7, 8]. However, anti-inflammatory treatments did not relieve pain in patients with neuropathies [9, 10], raising the possibility that additional cellular mechanisms or interactions might influence neuroimmune responses and pain hypersensitivity in neuropathic animals.

Recent evidence suggests that neuropathic pain is related to demyelination, which is the loss or damage of the myelin sheath that lines the nerve fibers, causing impaired nerve transmission [11]. Frequently at the basis of demyelination, there is a not resolved chronic inflammation process [12]. Microglia can communicate with neurons through the release of several mediators, such as growth factors, cytokines, and neurotrophins, or through direct contact [13, 14], making glia–neuron interaction a novel target for novel therapies for demyelinating diseases [15].

Posttranscriptional processing of gene expression and RNA stabilization of inflammatory proteins play a fundamental role in the regulation of pro-inflammatory response, and even though studies indicate the key role of posttranscriptional regulation of gene expression in neurodegenerative diseases [16], RNA processing remains poorly investigated in MS and MS-related symptoms. The embryonic lethal abnormal vision (ELAV)/Hu proteins represent a family of RNA binding proteins (RBPs) that have been originally identified as the targets of autoantibodies found in patients with paraneoplastic encephalomyelitis [17]. There are four mammalian ELAV RBPs encoded by separate genes, HuB, HuC, HuD, and HuR, with the biological function to posttranscriptionally control gene expression [18]. HuR is an ubiquitous protein that, besides neurons, is expressed in activated microglia [19, 20], and it is involved in cytoplasmic stabilization and/or enhancement of translation of mRNA of pro-inflammatory mediators, representing an important regulator of inflammatory and immune processes [21]. A pathological role for HuR in neuroinflammatory disorders has emerged, and its involvement in the pathogenesis of neurodegenerative and demyelinating disease has been postulated [22]. HuB, HuC, and HuD are neuron-specific proteins involved in neuronal differentiation, axonal outgrowth, and neuronal integrity [23]. HuD, the best-studied member of neuronal ELAVs (nELAVs), has been shown to promote neuroprotection and regeneration of peripheral nerves through the regulation of brain-derived neurotrophic factor (BDNF) and GAP43 mRNAs [24, 25].

Based on literature data, HuR overexpression could promote inflammatory phenomena, which might lead to neurotoxic consequences. It has also been described that, when all ELAV proteins are expressed in the same neuron, there is an interplay between nELAVs and HuR. Indeed, the appearance of nELAVs in neuroblasts downregulated HuR expression, inducing neuronal differentiation [26] and indicating that these proteins act in concert. Thus, in addition to direct proinflammatory activity, an aberrant HuR content might imbalance HuD/HuR protein ratio, promoting demyelination and neurodegeneration. All this evidence makes HuR and HuD potential targets to attenuate demyelination in neuropathic pain states. Verification of this hypothesis has been pursued in mouse models of MS EAE through an ASO-based approach to control HuR-mediated and HuD-mediated posttranscriptional regulation of gene expression.

## Materials and Methods

### Animals

Experiments for MOG_35-55_ EAE model were performed on CB57BL/6 female mice (20–25 g, 10 weeks old), and for PLP_139–151_, EAE were performed on SJL female mice (18–20 g, 10 weeks old) from Envigo (Varese, Italy). Experiments for SNI were performed on male CD1 mice (24–26 g, 4 weeks old) from Envigo (Varese, Italy). Animals have been housed in the Ce.S.A.L. (Centro Stabulazione Animali da Laboratorio, University of Florence) vivarium and used 1 day after their arrival. Mice have been housed in standard cages, with four animals per cage, kept at 23 ± 1 °C with a 12-h light/dark cycle, light on at 7 a.m., and fed with a standard laboratory diet and tap water ad libitum. The cages were placed in the experimental room for 24 h before behavioral testing for acclimatization, and all tests were conducted during the light phase.

Mice were sacrificed by cervical dislocation for removal of the spinal cord for in vitro analyses. The number of animals per experiment was based on a power analysis [27], and all tested groups comprised eight animals.

### Antisense Oligonucleotide Administration

Phosphorothioate oligonucleotides (ODNs) (resistant to exonuclease-mediated degradation) were used (Tib Molbiol, Genoa, Italy). The antisense ODN (ASO) against HuR was the following: 5’–A*T*AACCATTAGACATT*G*T–3, where the asterisks indicate the phosphorothioate phosphate groups. The ASO against HuD was the following: 5′–G*T*TCTGGAGCCTCATC*T*T–3′ where the asterisks indicate the phosphorothioate phosphate groups. A 18-mer fully degenerate ODN (dODN), where each base was randomly G, C, A, or T, was used as the control ODN treatment. To enhance both uptake and stability, ODNs were preincubated at 37 °C for 30 min with 13 µM DOTAP (Sigma, Milan, Italy), an artificial cationic lipid. ODNs were administered at the dose of 1 nmol/mouse on the basis of previous results [28, 29]. The experimental protocol to test the effect of ASO on behavioral and in vitro tests included 3 control groups: dODN (as control ODN), vehicle (DOTAP 13 µM), and saline.

### Intranasal Administration

For intranasal (i.n.) administration, mice were slightly anesthetized by 2% isoflurane inhalation and placed in a supine position. A 5-µL aliquot of DOTAP, dODN, or ASO has slowly dropped alternatively to each nostril with a micropipette tip. A total of 10 µL of solution containing 1 nmol of ODNs was delivered to mice. In the EAE model, treatments performed before immunization might neutralize the effect of an artificial disease induction and do not always address the therapeutic potential of drugs for patients at risk. HuR or HuD silencing was, thus, produced starting from day 14 of immunization, corresponding to the first disease peak. To achieve the protein knockdown, mice received a single i.n. injection every 2 days. The spinal cords were removed on day 30 post-immunization. In the SNI model, treatments were delivered every 2 days starting from day 3 post-surgery, and the spinal cords for in vitro tests were removed on day 10.

### EAE Induction

Sex-related differences in innate and adaptive immune responses may account for a prevalent female susceptibility to develop EAE symptoms [30]. Myelin oligodendrocyte glycoprotein (MOG_35–55_) was used to induce chronic progressive EAE in CB57BL/6 female mice [31], and myelin proteolipid protein (PLP_139–151_) was used to induce relapsing–remitting EAE in SJL female mice [32]. Animals were immunized subcutaneously (s.c.) in the flanks and at the base of the tail with a total of 200 µg of PLP_139–151_ or MOG_35–55_ peptide (synthesized by EspiKem Srl., University of Florence, Italy) per animal emulsified in complete Freund adjuvant (CFA; Sigma, Milan, Italy) supplemented with 4 mg/ml of Mycobacterium tuberculosis (strain H37Ra; Difco Laboratories, Detroit, Michigan, USA). Control mice received CFA without antigen. Immediately thereafter, and again 48 h later, all mice received an intraperitoneal (i.p.) injection of 500 ng Pertussis Toxin (Sigma, Milan, Italy) in 100 µl phosphate buffer saline (PBS). The general health and body weights of all mice were assessed prior to immunization and once daily thereafter in a blinded manner until the completion of the study. Locomotor coordination and nociceptive threshold were analyzed before onset and regularly during the course of the disease.

### Spared Nerve Injury (SNI)

Behavioral testing was performed before surgery to establish a baseline for comparison with postsurgical values. Mononeuropathy was induced by SNI, as previously described [33]. Mice were anesthetized with a mixture of 4% isoflurane in O_2_/N_2_O (30:70 *v/v*) and placed in a prone position. The right hind limb was slightly elevated, and a skin incision was made on the lateral surface of the thigh. The sciatic nerve was exposed at mid-thigh level distal to the trifurcation and freed of connective tissue; the three peripheral branches (sural, common peroneal, and tibial nerves) of the sciatic nerve were exposed without nerve structures. Both tibial and common peroneal nerves were ligated with microsurgical forceps (5.0 silk, Ethicon; Johnson & Johnson Intl., Brussels, Belgium) and transected together. The sural nerve was carefully preserved by avoiding any nerve stretch or contact with surgical tools. Muscle and skin were closed in two distinct layers with silk 5.0 sutures. Intense, reproducible, and long-lasting thermal and mechanical allodynia-like behaviors are measurable in the non-injured sural nerve skin extensions. The sham procedure consisted of the same surgery without ligation and transection of the nerves.

### Behavioral Testing

Animals were habituated to the testing environment daily for at least 2 days before baseline testing. To evaluate the onset and progression of pain hypersensitivity, neuropathic mice were monitored 7, 10, 14, 17, and 21 days after surgery. Nociceptive responses to mechanical and thermal stimuli were measured every 30 min for 3 h before and after nerve surgery. Each mouse served as its own control, the responses being measured both before and after surgery. EAE mice were monitored 3, 7, 10, 14, 18, 20, 25, 28, and 30 days after immunization. All testing was performed with a blind procedure.

#### Von Frey Test

Mechanical allodynia was measured by using Dynamic Plantar Aesthesiometer (Ugo Basile, Bologna, Italy), as described [29]. The mice were placed in individual Plexiglas cubicles (8.5 cm *L* 3.4 cm *H* 3.4 cm) on a wire mesh platform and allowed to acclimate for approximately 1 h, during which exploratory and grooming activity ended. After that, the mechanical stimulus was delivered to the plantar surface of the hind paw of the mouse from below the floor of the test chamber by an automated testing device. A steel rod (2 mm) was pushed with electronic ascending force (0–5 g in 35 s). When the animal withdrew its hind paw, the mechanical stimulus was automatically withdrawn, and the force was recorded to the nearest 0.1 g. Nociceptive response for mechanical sensitivity was expressed as mechanical paw withdrawal threshold (PWT) in grams. PWT was quantified by an observer blinded to the treatment.

The mean PWT was calculated from six consecutive trials (each performed every 30 min) and averaged for each group of mice.

#### Hargreaves’ Plantar Test

The hermal nociceptive threshold was measured using Hargreaves’ device, as described [34]. Paw withdrawal latency in response to radiant heat (infrared) was assessed using the plantar test apparatus (Ugo Basile, Comerio, Italy). Each mouse was placed under a transparent Plexiglas box (7.0 × 12.5 cm^2^, 17.0 cm high) on a 0.6-cm-thick glass plate and allowed to acclimatize for 1–2 h before recording. The radiant heat source consisted of an infrared bulb (Osram halogen–bellaphot bulb; 8 V, 50 W) that was positioned 0.5 cm under the glass plate directly beneath the hind paw. The time elapsed between switching on the infrared radiant heat stimulus and the manifestation of the paw withdrawal response was measured automatically. The intensity of the infrared light beam was chosen to give baseline latencies of 10 s in control mice. A cut-off of 20 s was used to prevent tissue damage. Each hind paw was tested 2–3 times, alternating between paws with an interval of at least 1 min between tests. The interval between two trials on the same paw was at least 5 min. Nociceptive response for thermal sensitivity was expressed as thermal paw withdrawal latency in seconds. All determinations were averaged for each animal.

#### Clinical Disease Score

Clinical disease scoring of EAE and sham mice (control group) was undertaken once daily in a blinded manner to evaluate the severity and extent of motor function deficits using a 5-point scale with half-point gradations [35]. EAE scores were daily assessed: score 0, no obvious changes in motor functions; score 0.5, distal paralysis of the tail; score 1, complete tail paralysis; score 1.5, mild paresis of 1 or both hind legs; score 2, severe paresis of hind legs; score 2.5, complete paralysis of 1 hind leg; score 3, complete paralysis of both hind legs; and score 3.5, complete paralysis of hind legs and paresis of 1 front leg. EAE clinical disease was classified as a present by clinical scores ≥ 1, whereas clinical scores ≤ 0.5 were regarded as disease remission or absence. Mice reaching a score of 3.5 were excluded from the study.

#### Rotarod Test

The apparatus consisted of a base platform, a rotating rod with a diameter of 3 cm, and a non-slippery surface. The rod was placed at a height of 15 cm from the base. The rod, 30 cm in length, was divided into 5 equal sections by 6 disks. Thus, up to 5 mice were tested simultaneously on the apparatus, with a rod-rotating speed of 16 r.p.m. The integrity of motor coordination was assessed on the basis of the number of falls from the rod in 30 s, as described [36]. The performance time was measured before and regularly after immunization.

### Western Blot Analysis

The lumbar portion of the spinal cord was collected. Samples were homogenized in a homogenization buffer containing 25 mM Tris–HCl pH = 0.5, 25 mM NaCl, 5 mM EGTA, 2.5 mM EDTA, 2 mM sodium pyrophosphate (NaPP), 4 mM p-nitrophenylphosphate (PNFF), 1 mM Na_3_VO4, 1 mM phenylmethylsulfonyl fluoride (PMSF), 20 µg/ml leupeptin, 50 µg/ml aprotinin, and 0.1% SDS. The homogenate was centrifuged at 9000 × *g* for 15 min at 4 °C, the low-speed pellet was discarded. Protein concentration in the supernatant (whole cell lysate) was quantified using Bradford’s method (protein assay kit, Bio-Rad Laboratories, Milan, Italy). Membrane homogenates (20–50 mg) were separated on 10% SDS–PAGE and transferred onto nitrocellulose membranes (120 min at 100 V) using standard procedures. Blots were incubated overnight at 4 °C with specific antibodies against IBA1 (1:1000; sc–32725; RRID:AB_667733), HuD (1: 1000; sc–48421; RRID:AB_627766); HuR (1:1000, sc–5261; RRID:AB_627770), MBP (1:1000 sc–271524; RRID:AB_10655672), S100 α/ß (1:500, sc–58839; RRID:AB_2183338), GAP43 (1:500 sc–17790; RRID:AB_627660) (Santa Cruz Biotechnology Inc, Santa Cruz, CA), neurofilament H (1:1000 bs–0708R; RRID:AB_10855865) (Bioss, Boston, MA). Blot visualization (chemiluminescence detection system) and signal intensity quantification (ImageJ, Wayne Rasband, National Institute of Health, USA) were performed. The exposition and developing time used were standardized for all the blots. Several reports suggest that commonly used housekeeping proteins are not equally expressed across cell types and experimental conditions, and quantification normalization of signal intensity to total protein loading is preferred [37]. For each sample, the signal intensity was normalized to that of total protein stained by Ponceau S, and the acquired images were quantified using Image Lab software. Sham mice were used as a control group. Measurements in control samples were assigned a relative value of 1, and measurements were expressed as fold to control value.

### Immunofluorescence

Experiments were performed on the lumbar portion of the spinal cord. Samples have been fixed in formalin at 4% for 24 h, dehydrated in alcohol, included in paraffin, and finally cut into 20 μm sections (spinal cords) and 10 μm (sciatic nerve; dorsal root ganglia). Primary antibodies used were antibodies for microglia (CD11b) (1:100; bs–11127; RRID:AB_10856024), neurofilament H (1:100 bs–0708R; RRID:AB_10855865) (Bioss, Boston, MA), HuD (1: 100; sc–48421; RRID:AB_627766), HuR (1:100, sc–5261; RRID:AB_627770), MBP (1:100, sc–271524; RRID:AB_10655672), S100 α/ß (1:100, sc–58839; RRID:AB_2183338), and GAP43 (1:500 sc–17790; RRID:AB_627660). After rinsing in PBS containing 0.01% Triton–X–100, sections were incubated in secondary antibodies labeled with Invitrogen Alexa Fluor 488 (490–525, 1:400; RRID:AB_221544), Invitrogen Alexa Fluor 568 (578–603, 1:400; RRID:AB_2534072) (Thermo Fisher Scientific), and Cruz Fluor 594 (592–614, 1:400; RRID:AB_2847914) (Santa Cruz Biotechnology) at room temperature for 2 h. Sections were coverslipped using a Vectorshield mounting medium (Vector Laboratories, Burlingame, CA). A Leica DM6000B fluorescence microscope equipped with a DFC350FX digital camera with appropriate excitation and emission filters for each fluorophore was used to acquire representative images. Images were acquired with 35 to 340 objectives using a digital camera. The immunofluorescence intensity was calculated using Image J. The extent of colocalization was determined by calculating Mander’s overlap coefficient, as described [20].

### Evaluation of BBB Disruption

Under normal conditions, Evans Blue (EB) dye conjugates with serum albumin to form a large complex that is not able to cross an intact barrier. However, when BBB is disrupted, this complex can enter the CNS [38]. Thus, the level of BBB disruption was detected by quantitative measurement for Evans blue content, as previously described [20] on day 28 after immunization. Briefly, mice were intraperitoneally injected with 2% Evans blue solution at a dose of 5.0 ml/kg per mouse. The dye was allowed to circulate for 4 h, and the mice were subsequently anesthetized and perfused transcardially with saline to remove the Evans blue dye in the vascular system. The brain and lumbar spinal cord were immediately removed, and images were captured. Tissues were homogenized with 2.5 ml PBS and mixed with 2.5 ml 50% trichloroacetic acid to precipitate protein overnight at 4 °C. The samples were centrifuged for 30 min at 10,000 × g, and the supernatants were measured at 610 nm for the absorbance of Evans blue by using a MP96 spectrophotometer (Safas, Monaco). The Evans blue content was expressed as micrograms per gram of brain and lumbar spinal cord.

### Luxol Fast Blue (LFB) Myelin Staining

LFB is a commonly used method to visualize myelin through an optical microscope, and it is used to detect demyelination in the central nervous system. Spinal cord samples are fixed in formalin at 4% for 24 h, dehydrated in alcohol, included in paraffin, and finally cut into 20-μm sections and placed on a glass slide. Then, the slides are washed 2 times in PBSB–Tryton 0.3% and left to complete drying. After that, colorant LFB is added to the slides and put at 60° C for 4 h. Subsequently, slides are washed in EtOH 96% and H_2_O. The decolorization step has been made with lithium carbonate (0.1% in H_2_0) for 15 s and EtOH 70% for 30 s. The decolorization step has been made until there is a contrast between the blue of the white and the gray matter. Afterward, final washings are made with: EtOH 96% for 3 min, EtOH 100% for 3 min, and xylene for 5 min. Finally, we mounted the slides with a mounting medium and stored them at room temperature. The slides are viewed on an OLYMPUS BX63F.

### Determination of TNF-α, IL-1β, IL-6, and IL-17 from Plasma

Mice were put under general anesthesia by intraperitoneal injection of zoletil and xylazine cocktail. Blood samples were taken from the ventricle with a standard cardiac puncture method, centrifuged at 3000 × *g* for 10 min at 4 °C, and the plasma was collected. TNF-α, IL-1β, and IL-17 protein levels were evaluated by using noncompetitive sandwich ELISA (BioLegend e-Bioscience DX Diagnostic, Monza, Italy), following the supplier’s instructions. The absorbance was measured at 450 nm using a MP96 microplate reader spectrophotometer (Safas, Monaco), and cytokine levels were expressed as picograms per milliliter according to a standard calibration curve.

### Statistical Analysis

The data and statistical analysis comply with the recommendations on experimental design and analysis in pharmacology [39]. Results are expressed as mean ± SEM. Repeated measures of two-way analysis of variance (ANOVA) followed by the Bonferroni test were used to compare locomotor behavior and pain behaviors between neuropathic and sham mice. The Kruskal–Wallis nonparametric test followed by Dunn's test was used to compare score values between EAE-mice administered ASO, dODN, or vehicle. Score values comparison between EAE and control mice was assessed using the nonparametric Wilcoxon test. One-way ANOVA followed by the Tukey post hoc test was used to compare the remaining data. For behavioral assays, all tested groups comprised eight animals. For biochemical and histological experiments, sample sizes subjected to statistical analysis had five samples per group (*n* = 5), where *n* is equal to the number of independent values. The statistical significance criterion was *P* < 0.05. The computer program GraphPad Prism, version 9.0 (GraphPad Software Inc., San Diego, CA), was used.

## Results

### Effect of ELAV-Silencing on EAE Behavioral Phenotype

To evaluate the involvement of HuR in neuropathic pain and pain-related spinal demyelination, we used the EAE, an animal model of MS. The most widely used EAE models are the relapsing–remitting PLP_139–151_-induced and the chronic MOG_35–55_-induced models [40]. Both models are characterized by central neuropathic pain, extensive inflammation in the white matter, and demyelination, whereas in the chronic MOG model, axonal degeneration was more prevalent [41].

The role of HuR and HuD was evaluated by protein knockdown through an antisense-based strategy. We have recently demonstrated that intranasal (i.n.) anti-HuR ASO induced a spinal HuR protein knockdown comparable to that obtained after intrathecal (i.t.) administration [28]. On these bases, the ASOs were i.n. administered. The presence of an interplay between HuR and HuD was investigated by comparing the effect produced by anti-HuR ASO with those produced by the silencing of HuD (anti-HuD ASO), the neuronal isoform mainly involved in neuroregeneration and neuronal maintenance.

**Fig. 1:**
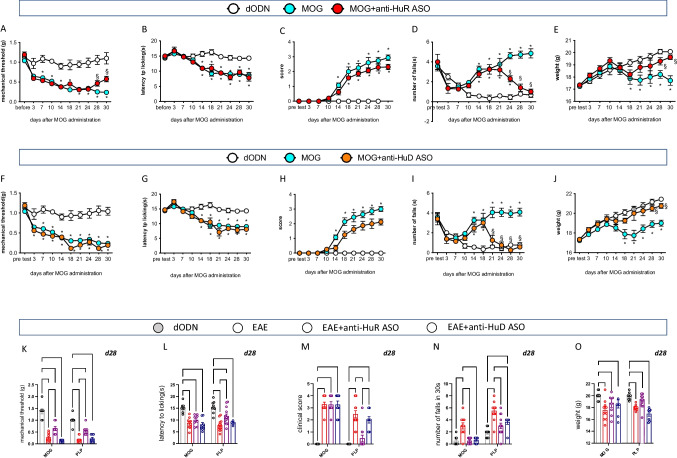
EAE phenotype following ELAV silencing. Time course of disease progression in the three experimental groups sham dODN, MOG dODN, and anti-HuR ASO on the MOG–EAE main behavioral parameters: mechanical hypersensitivity (von Frey filaments) (**A**), thermal hypersensitivity (plantar test) (**B**), clinical score (**C**), locomotor activity (rotarod test) (**D**), and body weight (**E**) (two-way ANOVA; *p < 0.05 vs sham dODN; §*p* < 0.05 vs MOG dODN). Evaluation of the behavioral phenotype following anti-HuD ASO over the 30-day period of the study on mechanical hypersensitivity (**F**), thermal hypersensitivity (**G**), clinical score (**H**), locomotor activity (**I**), and body weight (**J**) (two-way ANOVA; *p < 0.05 vs sham dODN; §*p* < 0.05 vs MOG dODN). Comparison of the effect of sham dODN, MOG dODN, anti-HuR ASO, and anti-HuD ASO on clinical signs observed in the MOG–EAE model (chronic multiple sclerosis) and in the PLP–EAE model (remitting relapsing multiple sclerosis) and on mechanical hypersensitivity (**K**), thermal hypersensitivity (**L**), locomotor activity (**M**), clinical score (**N**), and body weight (**O**) on day 28 post-immunization (d28) (two-way ANOVA *p < 0.05). Results are expressed as mean ± SEM. All tested groups comprised eight animals

MOG–EAE mice showed a significant reduction of the mechanical (Fig. 1A,F) and thermal threshold (Fig. 1B,G) in the hind paws from days 7 and 14 post-immunization, respectively, that persisted until day 30. Consistent with clinical symptoms in MS patients, EAE mice also showed motor disability, as indicated by the progressive increase of clinical score (Fig. 1C,H), and locomotor dysfunction, evaluated as impaired rotarod performance (Fig. 1D,I), from day 14 post-immunization up to day 30. EAE mice from day 14 showed a trend of body weight loss compared with sham mice, in which a constant and progressive increase in weight was recorded (Fig. 1E,J). Silencing of HuR reduced mechanical allodynia from day 28 (Fig. 1A) without affecting thermal hyperalgesia (Fig. 1B) and attenuated rotarod impairment (Fig. 1D). Even if not significant, a trend to attenuation of the disease clinical score was detected (Fig. 1C). Body weight loss was progressively attenuated by anti-HuR ASO, becoming statistically significant from day 28 (Fig. 1E). HuD silencing improved motor dysfunction (Fig. 1I), showed a trend toward a reduction of the clinical score with no significant effect (Fig. 3H), and attenuated body weight loss (Fig. 1J). Conversely to anti-HuR ASO, anti-HuD ASO did not modify pain hypersensitivity (Fig. 1F,G).

To better define the role of HuR and HuD in central neuropathic pain, results from MOG–EAE mice were compared with results from PLP–EAE mice. Conversely to MOG-EAE mice that showed a chronic and progressive trend of disability, PLP–EAE mice showed a relapsing–remitting profile with peaks of disability followed by remission. On day 28, there was a symptom recurrence that persisted until the completion of the study (Supplementary Fig. 1). Comparison of values recorded at 28-day post-immunization showed that anti-HuR ASO attenuated mechanical allodynia (Fig. 1K) and thermal hyperalgesia (Fig. 1L) in PLP-EAE mice, whereas anti-HuD ASO was ineffective on pain hypersensitivity (Fig. 1K,L). Furthermore, anti-HuR ASO improved both clinical score and locomotor disability (Fig. 1M,N) in the PLP–EAE model, resulting in more effectiveness in attenuating the overall EAE symptoms compared with the MOG–EAE model. These results are in line with the clinical efficacy of current therapies for MS in which a decrease in the relapse rate can be produced in relapsing–remitting patients, while efficacy in progressive forms is limited [42, 43]. In PLP–EAE mice anti-HuD ASO attenuated locomotor dysfunction (Fig. 1N), but it was ineffective on the clinical score (Fig. 1M), and further aggravated body weight loss (Fig. 1O).

### Anti-HuR ASO Increased Myelin, Neurofilament H, and GAP43 in Spinal Cord of MOG–EAE Mice

**Fig. 2:**
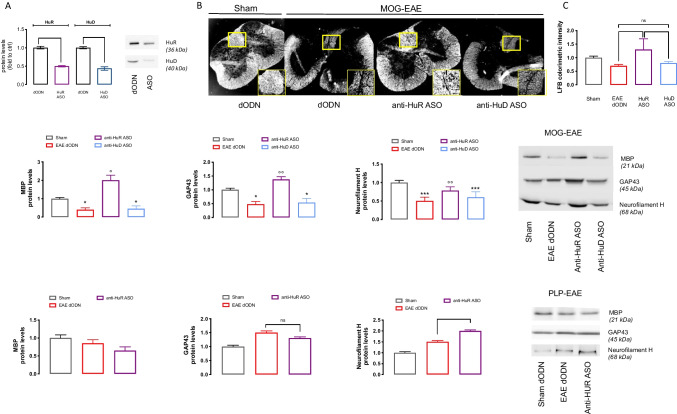
Role of HuR and HuD on demyelination and neurodegeneration. (**A**) Quantitative analysis of WB experiments performed on spinal cord tissue of naive mice showed decreased HuR and HuD expression after i.n. administration of corresponding ASO. (One-way ANOVA ***p < 0.001 vs dODN). Representative blots are reported. The signal intensity was normalized to that of total protein. Representative image (**B**) and quantitative analysis (**C**) of Luxol Fast Blue colorimetric assay of spinal cord tissue of sham, MOG-dODN, MOG-anti-HuR ASO, and MOG-anti-HuD ASO (white color correspond to myelin); scale bar = 100 µm (one-way ANOVA, *p < 0.05). Effect of anti-HuR ASO and anti-HuD ASO treatments in MOG–EAE mice on MBP (**D**), GAP43 (**E**), and neurofilament H (F) compared to the effects produced on PLP–EAE mice on the same marker (MBP, (**G**); GAP43, (**H**); neurofilament H, (**I**)). Results are expressed as mean ± SEM. Data are the mean of five individual experiments. Representative blots are reported. The signal intensity was normalized to that of total protein. Sham: dODN-treated sham mice

The efficacy of ASOs in knocking down protein levels within the spinal cord was confirmed by Western Blotting experiments showing the HuR and HuD protein reduction following corresponding ASO administration (Fig. 2A). Degenerate ODN (dODN) was used as control ODN. No difference was observed among naïve, vehicle- and dODN-treated groups (Supplementary Fig. 2).

The level of myelination within the CNS was detected in spinal cord samples of EAE mice by Luxol Fast Blue (LFB) staining. Representative images (Fig. 2B) and quantitative analysis (Fig. 2C) of spinal cord sections showed robust myelin loss in MOG–EAE mice that were significantly increased by anti-HuR ASO. These results were consistent with our previous data obtained in PLP–EAE mice [44]. In the spinal cord tissue of MOG–mice, a drastic reduction of myelin basic protein (MBP) was observed that was markedly up-regulated by anti-HuR ASO (Fig. 2D). Anti-HuD ASO had no effect on either LFB staining or MBP expression. HuR is expressed on oligodendrocyte progenitor cells (OPCs), as showed by colocalization with NG2, a marker of OPC. However, the anti-HuR ASO effect on myelination appeared not related to the promotion of OPC production since ASO did not alter the expression of both OPCs (NG2) and progenitor cells (nestin) content in spinal cord samples (Supplementary Fig. 3).

In addition to demyelination, neurodegeneration and axonal loss are a prominent feature in MOG–EAE mice, as demonstrated by the robust reduction of GAP43 (regeneration marker; Fig. 2E) and neurofilament H (axonal marker; Fig. 2F) protein level. Silencing of HuR increased the levels of neuro-regeneration markers up to control values, whereas anti-HuD ASO did not provoke any effect on all markers evaluated.

Results from MOG–EAE were compared to those obtained from PLP–EAE mice, a model of relapsing–remitting MS mainly characterized by spinal neuroinflammation and a lower degree of axonal loss that showed no significant modification of MBP (Fig. 2G), GAP43 (Fig. 2H), and neurofilament H (Fig. 2I) protein expression. Silencing of HuR produced a modest increase in neurofilament H expression and left unchanged the other two targets. Consistent with MOG–EAE data, anti-HuD ASO did not modify the expression of any markers evaluated.

### ELAV Silencing Inhibits BBB Permeability in EAE Mice

**Fig. 3:**
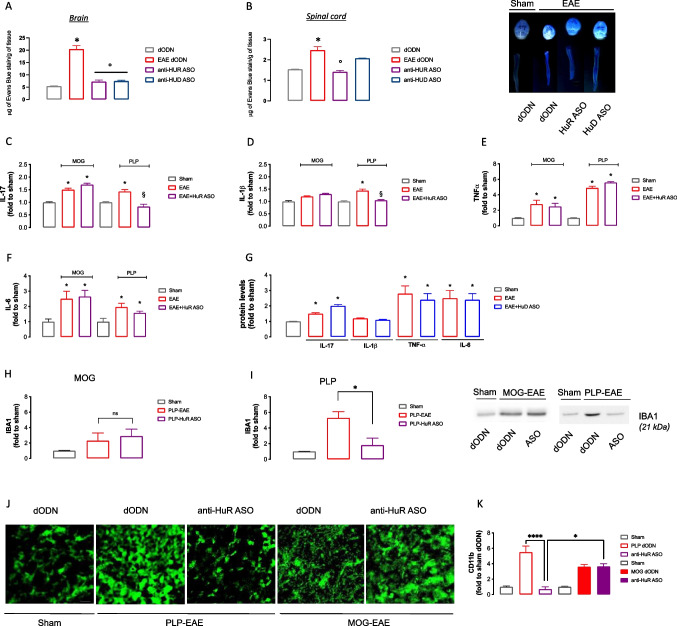
Effect of anti-HuR ASO on pro-inflammatory events. Anti-HuR ASO treatment inhibition of BBB permeability in the brain and spinal cord of EAE mice. The amount of extravasated Evans blue dye was quantified in the brain (**A**) and lumbar spinal cord (**B**) at 28 days after immunization. Representative pictures of the brain and spine from sham, MOG–EAE dODN, anti-HuR ASO, and anti-HuD ASO treated EAE mice are displayed. White color indicates extravasated Evans blue dye from blood vessels (one-way ANOVA *p < 0.05 vs sham, °*p* < 0.05 vs MOG dODN). Plasma levels of IL-17 (**C**), IL-1ß (**D**) TNF-alpha (**E**), and IL6 (**F**) in EAE mice and effect of anti-HuR treatment (one-way ANOVA *p < 0.05 vs sham; §*p* < 0.05 vs EAE). **G** Effect of anti-HuD ASO on circulating cytokines in MOG–EAE mice (one-way ANOVA *p < 0.05 vs sham). Sham: Dodn-treated sham mice. Expression of IBA1 protein in MOG–EAE (**H**) and PLP–EAE (**I**) mice and effect of anti-HuR ASO (one-way ANOVA *p < 0.05). Representative blots are reported. The signal intensity was normalized to that of total protein. **J** Representative images showing immunofluorescence staining for CD11b in the spinal cords of sham–dODN, EAE–dODN, EAE anti-HUR ASO-treated mice in the MOG and in the PLP model 28 days post-immunization. Scale bar = 100 µm. **K** Quantification of the immunofluorescence images; (two-way ANOVA *p < 0.05). All results are expressed as mean ± SEM. Data are the mean of five individual experiments

One of the major features of EAE and MS is the dysfunction of the blood–brain barrier (BBB). Endothelial cells express the IL-17 receptor, and IL-17 destroys the tight junctions of the BBB to facilitate the migration of CD4 + T cells in humans [45] and mice [46]. HuR can directly bind to the IL-17 mRNA, thus regulating IL-17 expression [47], and previous results showed the inhibition of BBB permeability by i.t. anti-HuR ASO in PLP–EAE mice [20]. These promising results encouraged us to investigate the effect of i.n. anti-HuR and anti-HuD ASOs on BBB leakage. Quantification analysis showed an increase of dye extravasation in the brain (Fig. 3A) and spinal cord (Fig. 3B) of MOG mice on day 28 post-immunization. Silencing of HuR largely decreased the EB content in both the brain and spinal cord, returning to values comparable with the level of the control mice. Anti-HuD reduced the dye content in the brain with an effect comparable to anti-HuR ASO. Conversely, in the spinal cord tissue, there was a trend toward a permeability reduction without reaching statistical significance. Representative images of the brain and spinal cord showed that the degree of EB leakage (white intensity) was increased in EAE mice.

### Effect of Anti-HuR and Anti-HuD ASOs on Inflammatory Mediators in EAE Mice

Quantification of the plasma level of cytokines showed an increase in IL-17 (Fig. 3C), TNF-⍺ (Fig. 3E), and IL-6 (Fig. 3F) in both MOG and PLP mice. IL-1β plasma levels were significantly increased only in the PLP-treated group (Fig. 3D). Treatment with anti-HuR ASO reduced the expression of IL-17, IL-1ß, and IL-6 in PLP mice with no effect on TNF-⍺ expression. No variation in circulating cytokine levels was observed in anti-HuR ASO-treated MOG–EAE mice. Anti-HuD ASO never modified the increased levels of circulating cytokines (Fig. 3G).

Although the broad range of neurological symptoms and stages of disease progression, a key hallmark of EAE and MS is neuroinflammation. In the spinal cord tissue of MOG–EAE mice (Fig. 3H) and, more abundantly, of PLP–EAE mice (Fig. 3I), an increased expression of the microglia marker IBA1 has been observed. IBA1 expression was reduced by anti-HuR ASO treatment in the PLP group, while it remained unmodified in MOG mice. To confirm these data, immunofluorescence experiments (Fig. 3J) and quantification analysis (Fig. 3K) showed a modestly increased expression of CD11b protein (a marker of the activated state of microglia) in MOG–EAE mice that were not modified by HuR silencing. Results were compared to those obtained from PLP mice that showed a much more intense increase of CD11b expression that was abolished to control levels by anti-HuR ASO.

### Effect of ELAV Silencing on Thermal and Mechanical Allodynia in Peripheral Neuropathic Pain

**Fig. 4:**
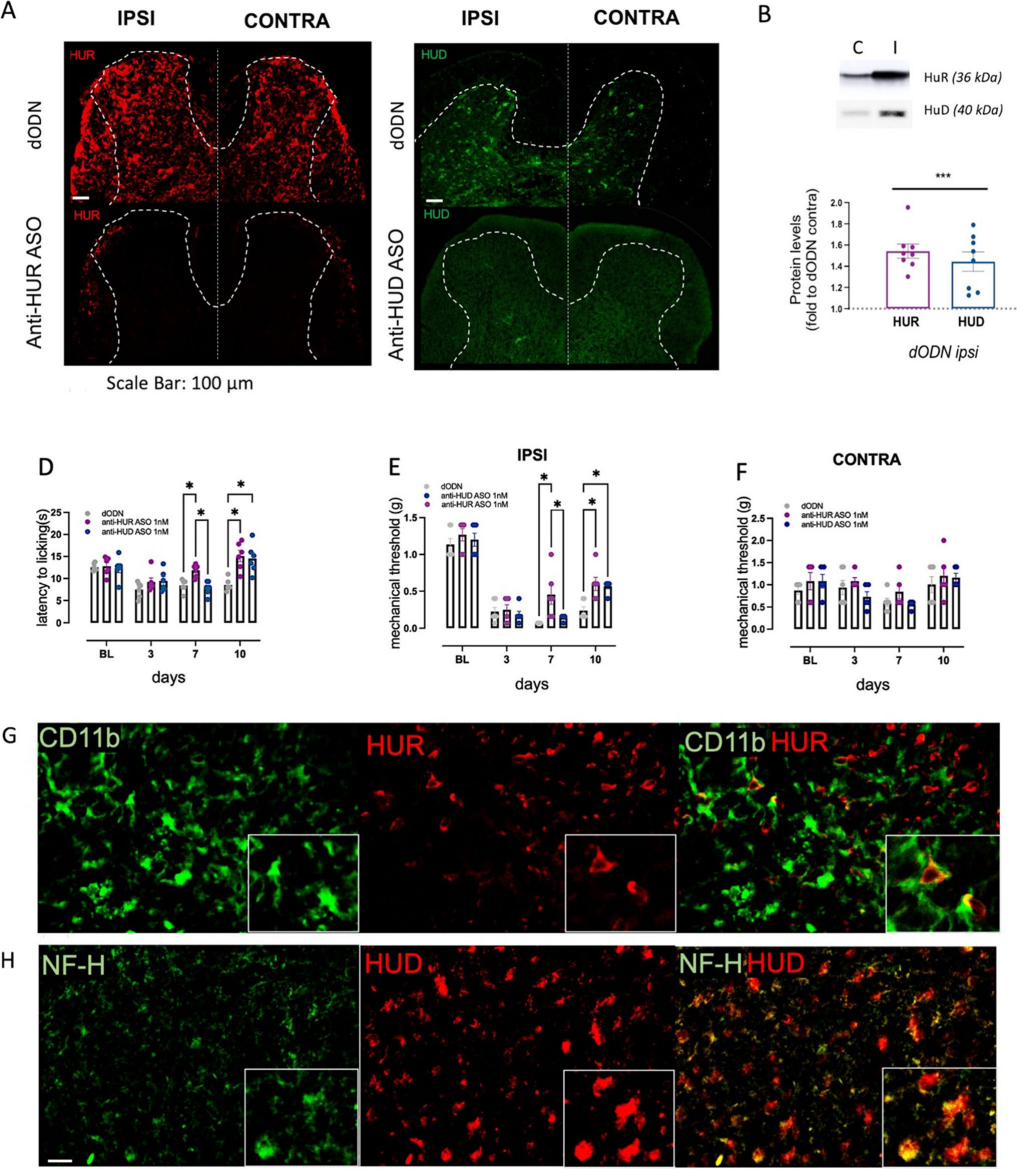
HuR silencing attenuated pain hypersensitivity in SNI mice. **A** Increase of HuR (red) and HuD (green) protein levels in the ipsilateral (injured) spinal dorsal horn of SNI mice compared to the contralateral (uninjured) side and downregulation by corresponding ASO (bottom images). Samples were collected 10 days after nerve ligation. Scale bar = 100 µm. **B** Quantitative analysis of western blotting experiments performed on spinal cord tissue of SNI mice showed increased HuR and HuD expression (one-way ANOVA *p < 0.05 vs dODN contra). Representative blots are reported. The signal intensity was normalized to that of total protein. In SNI mice, HuR and HuD silencing attenuated thermal allodynia (**D**) and alleviated mechanical allodynia in the ipsi hind paw (**E**), with no effect on contra hind paw (**F**), on days 7 and 10 after surgery (two-way ANOVA *p < 0.05). **G** Colocalization of HuR (red) with CD11b (green) and (H) colocalization of HuD (red) with neurofilament H (green) in the ipsilateral side of the spinal cord of SNI mice (Mander’s coefficient = 0.89). Results are expressed as mean ± SEM. For behavioral assays, all tested groups comprised eight animals. For biochemical and immunohistochemical assays, data are the mean of five individual experiments

Previous findings showed a spinal HuR overexpression induced by the spared nerve injury (SNI) procedure [28], a model of peripheral mononeuropathy. To better define the role of ELAV isoforms in the central events involved in neuropathic pain associated with demyelination, we compared results from EAE mice, characterized by central neuropathic pain, with those obtained in SNI, a model of monolateral peripheral neuropathic pain. The effects produced by i.n. ELAV silencing were investigated by comparing the nociceptive phenotype with the modulation of main markers at the central (spinal cord) and peripheral (sciatic nerve) level. In the ipsilateral (injured) side of the spinal cord tissue of SNI mice, increased immunostaining of HuR (Fig. 4A, red, upper panel) and HuD (Fig. 4A, green, upper panel) was detected in comparison with the contralateral (uninjured) side. These results have been confirmed with western blotting analysis (Fig. 4B). Silencing of HuR or HuD by i.n. administration of anti-HuR ASO (Fig. 4A, red, bottom) and anti-HuD ASO (Fig. 4A, green, bottom), respectively, reduced the corresponding protein expression.

Behavioral nociceptive tests showed that anti-HuR ASO reduced thermal (Fig. 4D) and mechanical (Fig. 4E) allodynia in SNI mice starting from 7 days after surgery, whereas anti-HuD ASO reduced pain hypersensitivity from post-surgical day 10. Both of ASOs did not affect the mechanical threshold on the contralateral side (Fig. 4F).

Immunofluorescence experiments performed on the external laminae of the spinal cord dorsal horn showed the colocalization of HuR with CD11b, a marker of activated state of microglia (Fig. 4G), whereas the neuronal ELAV HuD colocalized with neurofilament H (NF–H) (Fig. 4H), an axonal marker, confirming the cellular localization previously described in the literature [28, 29].

### HuR-Silencing Increased Spinal Myelin Content in SNI Mice

**Fig. 5:**
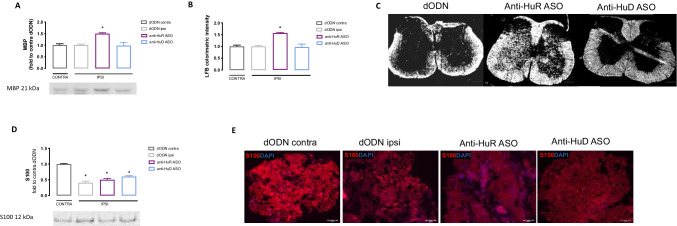
Effect of HuR silencing on myelination in SNI mice. **A** Silencing of HuR increased MBP, marker of myelin, in spinal cord of SNI mice (one-way ANOVA *p < 0.05 vs dODN ipsi) and lack of effect by HuD silencing. Representative blots are reported. The signal intensity was normalized to that of total protein. **B** Quantitative analysis of Luxol Fast Blue colorimetric assay and **C** representative image of spinal cord tissue of dODN, anti-HuR ASO, and anti-HuD ASO (white color corresponds to myelin, scale bar = 100 µm) (one-way ANOVA *p < 0.05 vs dODN ipsi). **D** Silencing of HuR and HuD did not alter the expression levels of S100 in sciatic nerve (one-way ANOVA, *p < 0.05 vs dODN contra). Representative blots are reported. The signal intensity was normalized to that of total protein. **E** Representative images of the expression of S100 (red) of ipsilateral side of sciatic nerve in SNI mice. Scale bar = 50 µm. Results are expressed as mean ± SEM. Data are the mean of five individual experiments

The level of myelination within the CNS was detected in spinal cord samples of SNI mice. No significant difference in MBP expression between the ipsilateral and contralateral sides of the spinal cord tissue of dODN-treated SNI mice was observed. I.n. anti-HuR ASO increased the levels of MBP, while no effect was detected following anti-HuD ASO administration (Fig. 5A). These results were consistent with LFB staining of the spinal cord sections. Quantitative analysis showed significantly increased staining in anti-HuR ASO-treated SNI mice compared to dODN mice (Fig. 5B), confirming a significant increase in the myelin content. Anti-HuD ASO mice did not show any modification in LFB staining compared to the control dODN group. Representative images showed a myelin staining (white marking) much more evident in the anti-HuR ASO group compared to the other groups (Fig. 5C).

The level of myelination within the peripheral nervous system was investigated in sciatic nerve samples of SNI mice. Myelination of Schwann cells was detected by quantifying the S100 protein levels, and a significant decrease in the expression of this protein was detected in the ipsilateral side of the dODN-treated SNI mice. Both i.n. anti-HuR and anti-HuD ASO groups did not alter S100 expression (Fig. 5D). Representative images of sciatic nerves showed the different intensity of S100 immunostaining between contra and ipsilateral sides of SNI control mice and the lack of effect by ASO treatments (Fig. 5E).

### Interplay Between HuR and HuD in the Promotion of Spinal Expression of MBP

**Fig. 6:**
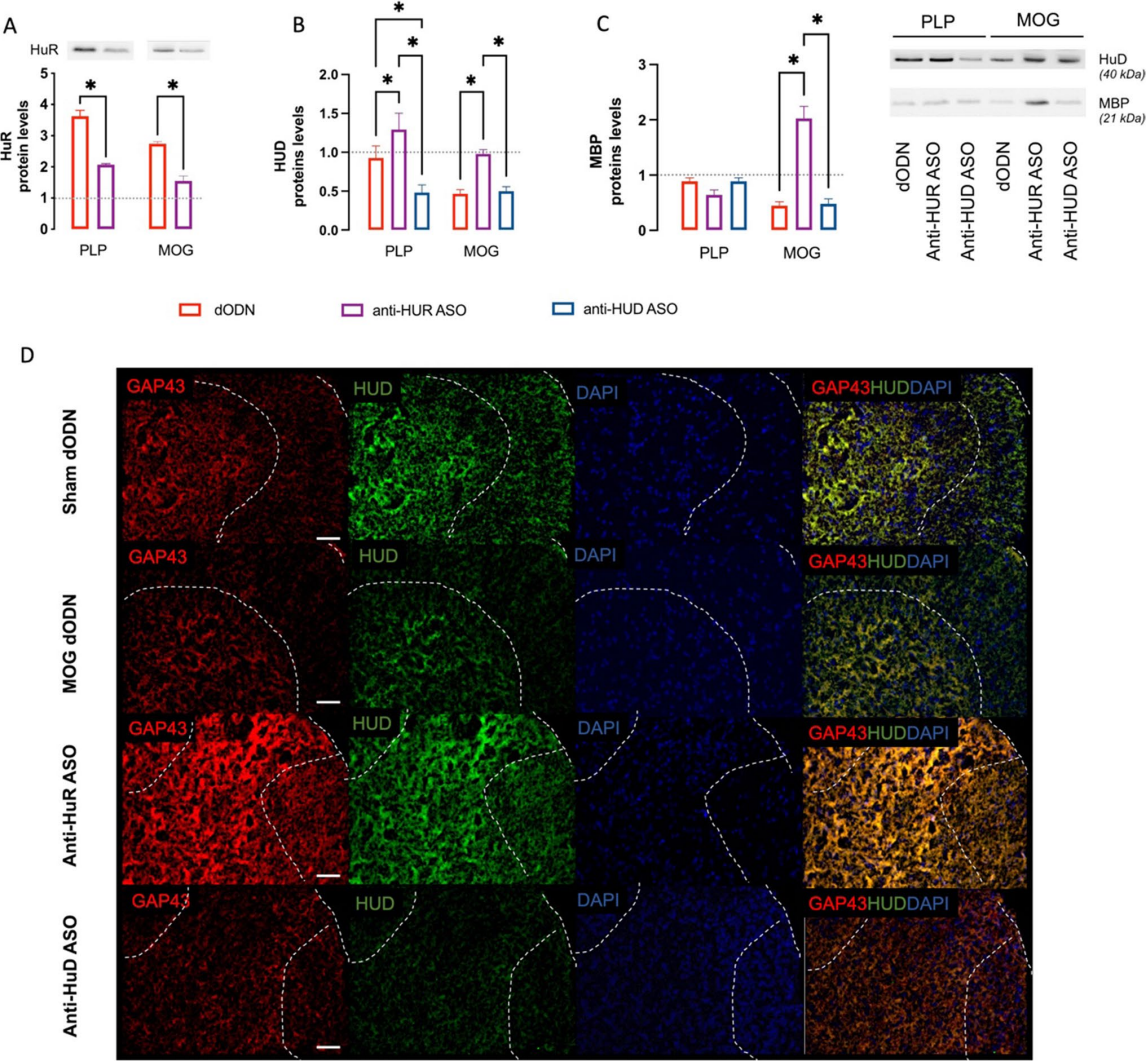
Interplay between HuR and HuD proteins. **A** HuR over-expression in PLP–EAE and MOG–EAE spinal cord samples. HuD (**B**) and MBP (**C**) expression in dODN, anti-HuR ASO, and anti-HuD ASO treated mice in the PLP and MOG EAE models (two-way ANOVA *p < 0.05). Dashed lines represent the control value obtained by dODN-treated PLP (1 ± 0.03) and MOG (1 ± 0.04) sham mice. Results are expressed as mean ± SEM. Data are mean of five individual experiments; EAE spinal cord samples were collected on day 28 post-immunization. Representative blots are reported. The signal intensity was normalized to that of total protein. **D** Colocalization of GAP43 (red) and HuD (green) in spinal cord dorsal horn section (dashed lines) from sham, MOG dODN, anti-HuR ASO, and anti-HuD ASO

To evaluate the presence of a connection between HuR and HuD, we investigated if HuR silencing might lead to a modification in the spinal HuD protein levels in conditions of neuropathic pain associated with the EAE models. Consistent with previous studies on PLP-EAE mice [20, 44], double-staining immunofluorescence experiments performed on the external laminae of the spinal cord dorsal horn from MOG-EAE mice confirmed the neuronal localization of ELAV HuD and the neuronal and microglial localization of HuR (Supplementary Fig. 4). HuR was upregulated in EAE models (Fig. 6A), as well as in SNI mice (Fig. 4), indicating HuR increase as a common event in neuropathic pain conditions. Conversely, HuD showed a different pattern of expression, being unaltered in PLP–EAE, drastically downregulated in MOG–EAE mice (Fig. 6B), and upregulated in SNI (Fig. 4). In both PLP–EAE and MOG–EAE models, anti-HuR ASO downregulated HuR expression (Fig. 6A), while it increased HuD expression (Fig. 6B). As expected, HuD protein levels were further reduced by treatment with anti-HuD ASO (Fig. 6B).

Anti-HuR ASO also differently influenced MBP expression in the models investigated. A robust increase of the myelin marker was detected in the MOG–EAE mice, the model in which HuD was drastically upregulated by HuR silencing, whereas no significant effect was observed in PLP-EAE samples in which HuD was unaltered (Fig. 6C).

To evaluate if the neuroprotective effect of the anti-HuR ASO treatment was mediated by HuD overexpression, we conducted double-staining immunofluorescence experiments. Micrographs confirmed the GAP43 (red) and HuD (green) reduced expression in MOG–EAE mice and their overexpression following anti-HuR ASO treatment. Merge images showed an abundant localization of HuD in GAP43-expressing cells (Fig. 6C), confirmed by quantification analysis (anti-HuR ASO Mander’s coefficient 0.89 ± 0.03).

## Discussion

Neuropathic pain represents the most difficult-to-treat pain condition in MS patients. The unmet need for effective treatments has led to increasing attempts to define the complex pathophysiology of neuropathic pain. Recent evidence related neuropathic pain to demyelination, a condition that is usually related to unresolved neuroinflammation. Thus, targeting demyelination appears a promising disease-modifying strategy to address neuropathic pain progression rather than merely suppress symptoms. In the present study, we investigated the involvement of posttranscriptional regulation of gene expression in demyelination-related pain hypersensitivity in mouse models of MS by focusing on the RNA-binding proteins (RBP) HuR and HuD. We observed that restoration of an aberrant expression of HuR counteracted demyelination and neurodegeneration and attenuated clinical symptoms.

Spinal cord sections of MOG–EAE mice (model of chronic MS) showed marked demyelination that was largely improved by i.n. anti-HuR ASO treatment. These results are consistent with previous findings on PLP–EAE mice (model of relapsing–remitting MS) treated intrathecally with an anti-HuR ASO [44]. I.n. anti-HuR ASO ameliorated mechanical allodynia and attenuated motor disability in both PLP–EAE and MOG–EAE mice. MS-associated neuropathic pain develops as a direct or indirect result of demyelinating lesions in the brain and spinal cord in areas involved in pain perception [48, 49], highlighting a functional relation of spinal HuR overexpression to neuropathic pain and spinal myelination. Indeed, the positive effects of demyelination temporally coincided with the attenuation of mechanical allodynia and motor disability, suggesting that the control of neuropathic pain might underly a protective activity toward demyelination.

Myelin restoration can be achieved by improving myelination as well as by dampening pro-demyelinating processes mainly consisting of aberrant immune and inflammatory responses. Considering that in MS patients inflammation declines with disease duration while neurodegeneration proceeds and activated microglia persists in all lesions in progressive MS, using the MOG–EAE model there was the need to answer the question of anti-HuR ASO effects resulted from a direct mechanism on myelination or indirectly through a central immune/inflammatory suppression. ASO reduced the BBB leakage in the brain and spinal cord during MOG–EAE in the absence of any significant reduction of circulating pro-inflammatory cytokines. Consistent with our findings, a recent clinical study showed a reduced HuR level in peripheral blood mononuclear cells from MS patients compared with the healthy subject [50]. By comparing MOG–EAE and PLP–EAE mice, spinal activated microglia were more abundant and more intensively reduced by anti-HuR ASO in the relapsing–remitting model. Conversely, the chronic model showed a lower degree of microglia activation that remained almost unmodified by ASO treatment. These data are consistent with the clinical observation of an inflammation decline from the relapsing–remitting MS to the progressive MS and let us hypothesize that an immune/inflammatory suppression, while relevant for the PLP–EAE model, is not prominently involved in the ASO-induced remyelinating and pain-relieving activity in the chronic model. HuR is ubiquitously expressed, and it has been detected in activated microglia as well as in neurons in the spinal cord of PLP-EAE mice [44]. The same cellular localization was also observed in MOG-EAE spinal cord. Thus, the protective effect toward demyelination in MOG–EAE mice can be produced, at least in part, through a neuron-mediated mechanism. HuR mRNA exists as three alternatively polyadenylated variants: a 1.5-kb mRNA isoform primarily expressed in the testes, a 2.4-kb mRNA isoform that is ubiquitous and represents the predominant transcript variant expressed in most mammalian tissues and found in the majority of cell types, and a 6.0-kb isoform expressed exclusively in neuron [51]. The HuR mRNA isoforms differ in the length of their 3′-UTRs, while they are identical in 5’-UTR and encode the exact same protein product. Of note, the 6.0-kb neuron-specific mRNA isoform is inherently less stable and produces less HuR protein than the ubiquitous 2.4-kb mRNA. Furthermore, nELAVs, as well as HuR itself, can bind at the 2.4-kb mRNA polyadenylation site and when overexpressed can affect alternative polyadenylation to generate an extended HuR 3′-UTR that is translationally suppressed [26]. When all ELAV members are expressed in the same neuron, an interplay between HuR and nELAVs in neuronal differentiation in vitro has been demonstrated, by which the appearance of nELAVs in neuroblasts downregulates HuR expression [26]. We, thus, hypothesized that the ASO-induced HuR knockdown might allow an nELAV overexpression that might promote neuroprotection. HuD, the best-studied member of the nELAVs, has shown a role in promoting neuroprotection in neuropathies. HuD-dependent GAP–43 [29, 52] and BDNF [24] increase has been shown in the DRG and axons during nerve regeneration in peripheral neuropathy models and growing evidence associates mRNAs HuD target to neurological and neurodegenerative disorders [23, 53, 54]. Thus, we investigated the presence of a connection between HuR and HuD. While both models showed spinal HuR overexpression, they showed a different pattern of HuD expression. MOG mice showed a drastic loss of HuD protein in the spinal cord that temporally coincided with HuR overexpression. These events were also concomitant with myelin loss and decreased expression of axonal (neurofilament H) and neuroregeneration (GAP43) markers. HuR silencing restored basal levels of HuD protein as well as neurofilament H and GAP43. Dampening axonal degeneration could extend the period during which axons could be remyelinated, thus favoring myelin restoration. Since GAP43 [29] and neurofilament H [55] are among the main mRNA targets of HuD, these findings let us hypothesize that the neuroprotective activity produced by anti-HuR ASO in chronic EAE could underlie an indirect mechanism related to the restoration of HuD expression. The 2-week period of repeated treatment needed to ease pain hypersensitivity in MOG-EAE mice further supports the hypothesis of a long-lasting indirect mechanism.

PLP-EAE mice showed unaltered HuD, GAP43, and neurofilament H levels that remained almost unmodified by anti-HuR ASO treatment, indicating that in this model the HuD-mediated spinal neuroprotection does not appear prominently activated. PLP–EAE is a model that mirrors the early stages of MS, in which neuroinflammation is a main feature. Previous results showed that intrathecal HuR ASO in PLP-EAE mice induced myelin restoration related to the attenuation of microglia-mediated neuroinflammation as well as a pain-relieving activity that appeared starting from 1 week of treatment and persisted up to the end of experimentation [44]. To better define the role of HuR in CNS events involved in demyelination-associated neuropathic pain, the effect of i.n. anti-HuR ASO on spinal myelination was investigated in SNI mice, a model of peripheral mononeuropathy characterized by increased spinal HuR levels [28]. Consistent with literature data, we observed an overexpression of spinal HuR in SNI mice, comparable to that found in EAE mice, with a prominent microglial localization, suggesting HuR upregulation as a common trait for neuropathic pain conditions. Anti-HuR ASO treatment inhibited microglia-mediated spinal neuroinflammation and attenuated the nociceptive phenotype [28]. Even if demyelination was not detected in spinal cord sections, HuR knockdown largely increased the spinal myelin content. In mice with a sciatic nerve lesion, neuropathic pain is sustained by the recruitment of monocytes/macrophages at the peripheral level and by a microglia-mediated spinal pro-inflammatory response [56, 57]. Thus, consistent with the role of HuR in promoting a spinal neuroinflammatory response through microglia activation described in human and animal models [19, 28], the attenuation of microglia-mediated neuroinflammation appears as a plausible mechanism for the positive effect on spinal myelination by HuR silencing.

These findings highlight a multitarget activity of HuR that allows the anti-HuR ASO to control neuroinflammation through microglia-mediated mechanisms and to attenuate neurodegeneration through neuronal mechanisms mediated by the overexpression of HuD. Thus, besides the microglia-mediated anti-neuroinflammatory activity, the activation of HuD-mediated pathways represents an additional mechanism for myelin protection that is promoted when neurodegeneration represents a prominent event. Anyway, both mechanisms are related to myelin protection, making anti-HuR ASO an innovative approach to attenuate demyelination-associated neuropathic pain in early and late disease states.

A further improvement for neuropathic pain therapy is represented by the i.n. delivery route. Even though the clinical efficacy of ASO-based therapies has been assessed, the difficulty to deliver ODNs to the CNS because of their poor capability to cross the BBB limits their application in pathological conditions that require CNS penetration. The i.n. drug delivery represents a simple, noninvasive, allowing self-medication in patients, route of administration that facilitates a rapid achievement of therapeutic drug concentrations in the brain and cerebrospinal fluid. This way could help avoid unpleasant delivery methods (i.e., intrathecal) currently required for ASO-based therapies. The wide interest aroused by this delivery route is demonstrated by 730 clinical trials underway in the US alone using i.n. administration [58].

## Conclusions

In conclusion, spinal HuR overexpression was observed in both chronic and relapsing–remitting EAE mice and i.n. administration of an anti-HuR ASO attenuated pain hypersensitivity. This effect was induced by promoting myelin restoration through either HuD-mediated neuroprotective phenomena in chronic EAE mice or by dampening pro-inflammatory responses mediated by spinal microglia activation in relapsing–remitting EAE mice. Present findings could represent an important step to better understand the pathophysiological role of post-transcriptional regulation of gene expression in demyelination-associated neuropathic pain conditions and make targeting HuR an innovative and promising step toward disease-modifying strategies for the management of neuropathic pain states.

## Supplementary Information

Below is the link to the electronic supplementary material.

Supplementary file1 (DOCX 18784 KB)

## Data Availability

The data that support the findings of this study are available from the corresponding author upon reasonable request.
