# Supplementary material for: Posttranscriptional Regulation of Gene Expression Participates in the Myelin Restoration in Mouse Models of Multiple Sclerosis: Antisense Modulation of HuR and HuD ELAV RNA Binding Protein
Source: Mol Neurobiol. 2023 Jan 25;60(5):2661–77. doi: 10.1007/s12035-023-03236-8 (PMC10039839; doi:10.1007/s12035-023-03236-8)
Supplement: Supplementary file 1 — Supplementary file1 (DOCX 18784 KB) [file 12035_2023_3236_MOESM1_ESM.docx]

**Myelin restoration by antisense modulation of HuR and HuD ELAV RNA binding protein expression in mouse models of multiple sclerosis**

Vittoria Borgonetti, PhD, Nicoletta Galeotti, PhD

Department of Neuroscience, Psychology, Drug Research and Child Health (NEUROFARBA), Section of Pharmacology, University of Florence, Viale G. Pieraccini 6, 50139 Florence, Italy.

**SUPPLEMENTARY MATERIAL**


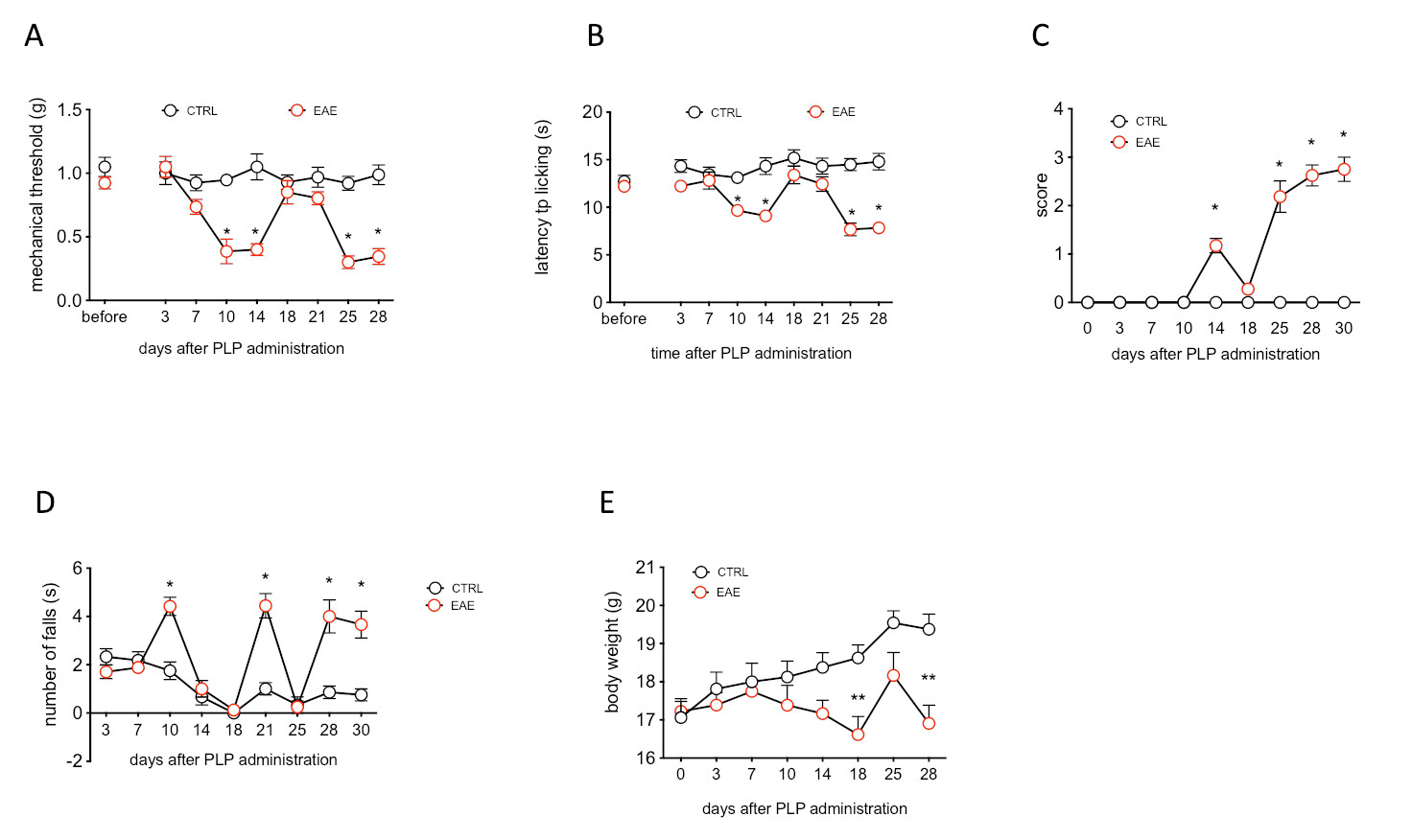


**Supplementary Fig. 1. Disease progression of PLP_139-151_-EAE mice**. Time course study of mechanical (A) and thermal (B) allodynia in PLP-EAE mice showed a relapsing-remitting profile with a first peak of disease on days 10-14 and a second peak from day 25. (C) Clinical disease score of PLP-EAE mice in comparison with control mice (CTRL) showed a relapsing-remitting profile. The first peak of disease was on day 14 and the second peak started from day 25. (D) Progression of locomotor impairment in PLP-EAE mice evaluated by the rotarod performance. A first peak of disability was detected on day 10, a second peak on day 21 and a third peak from day 28. (E) Time-course evaluation of body in EAE mice showed a progressive loss of weight with a first peak on day 18, followed by a recover and then a second peak from day 28. *P<0.05, **P<0.01, ***P<0.001 vs CTRL mice.


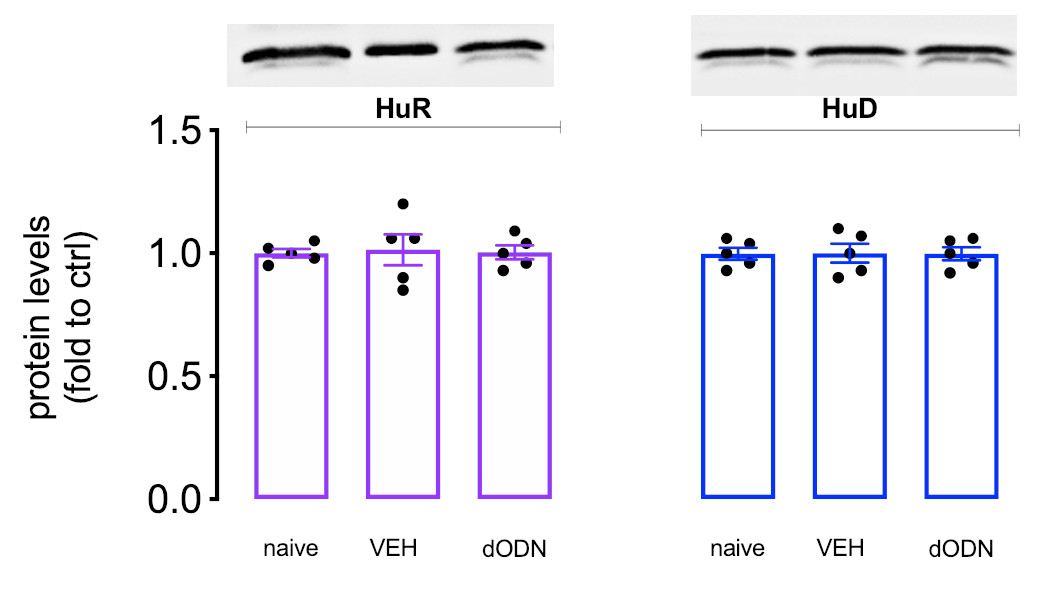


**Supplementary Figure 2. HuR and HuD expression in naïve, vehicle– and dODN–treated groups**. No difference in HuR and HuD levels was observed in VEH– and dODN–treated groups in comparison with naïve mice. VEH: vehicle (DOTAP 13 µM); dODN: degenerate ODN. Results are expressed as mean ± SEM. Data are mean of five individual experiments. Representative blots are reported. The signal intensity was normalized to that of total protein.


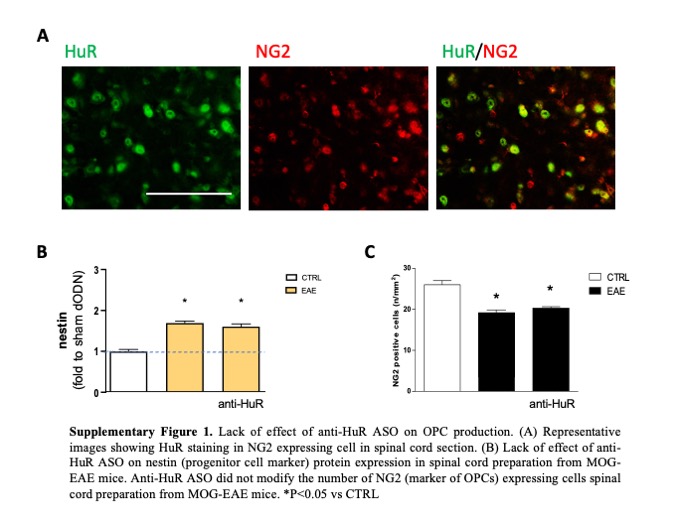


**Supplementary Fig. 3. Lack of effect of anti-HuR ASO on OPC production**. (A) Representative images showing HuR staining in NG2 expressing cell in spinal cord section. (B) Lack of effect of anti-HuR ASO on nestin (progenitor cell marker) protein expression in spinal cord preparation from MOG-EAE mice. Anti-HuR ASO did not modify the number of NG2 (marker of OPCs) expressing cells spinal cord preparation from MOG-EAE mice. *P<0.05 vs CTRL.


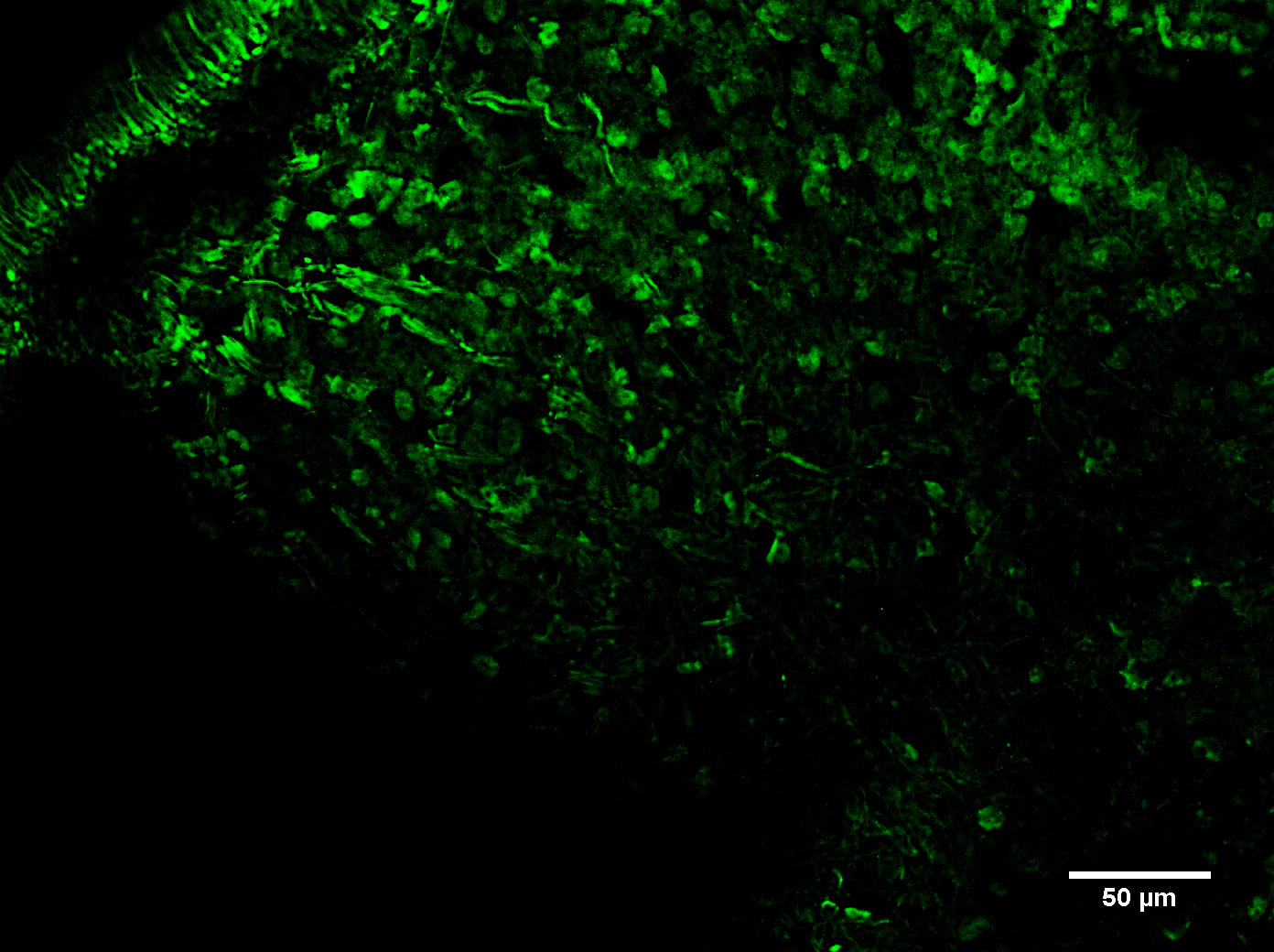

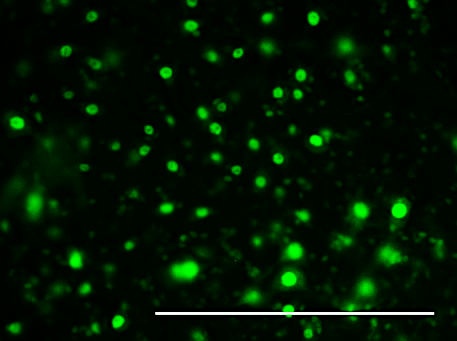

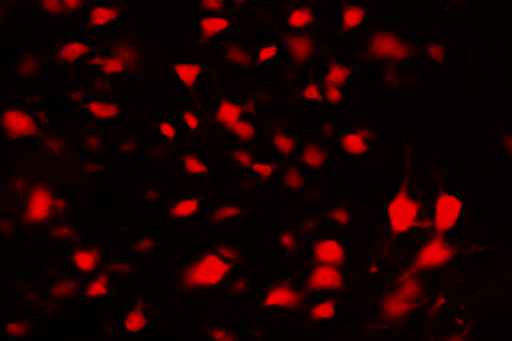

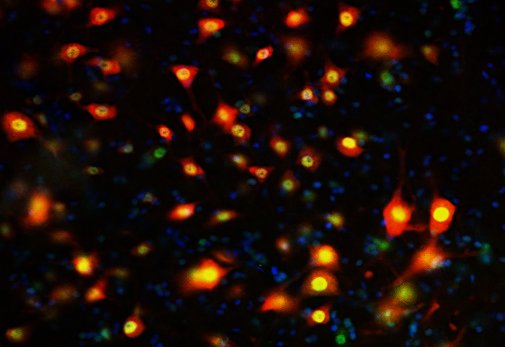


HuD

NeuN

MERGE

CD11


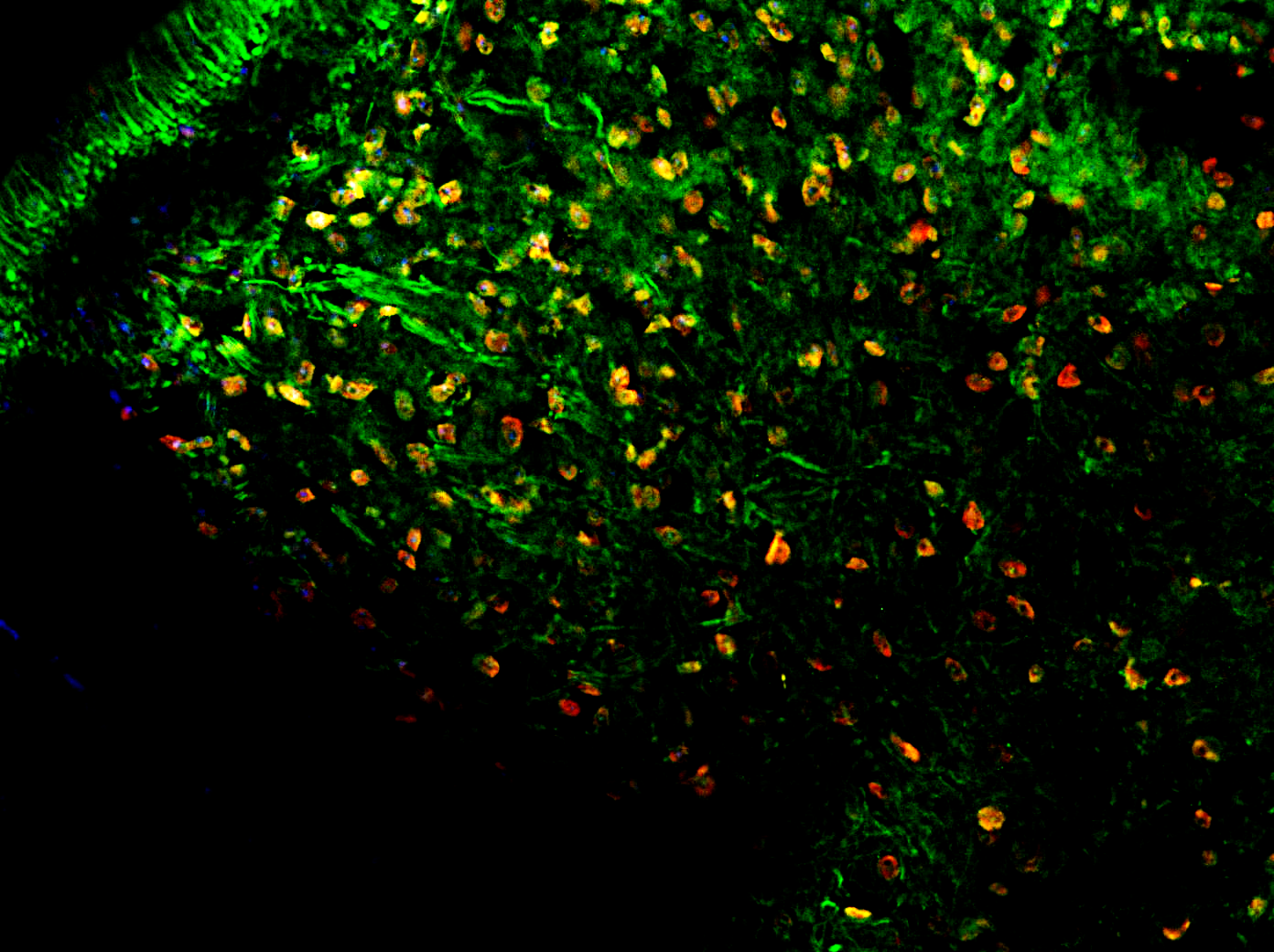

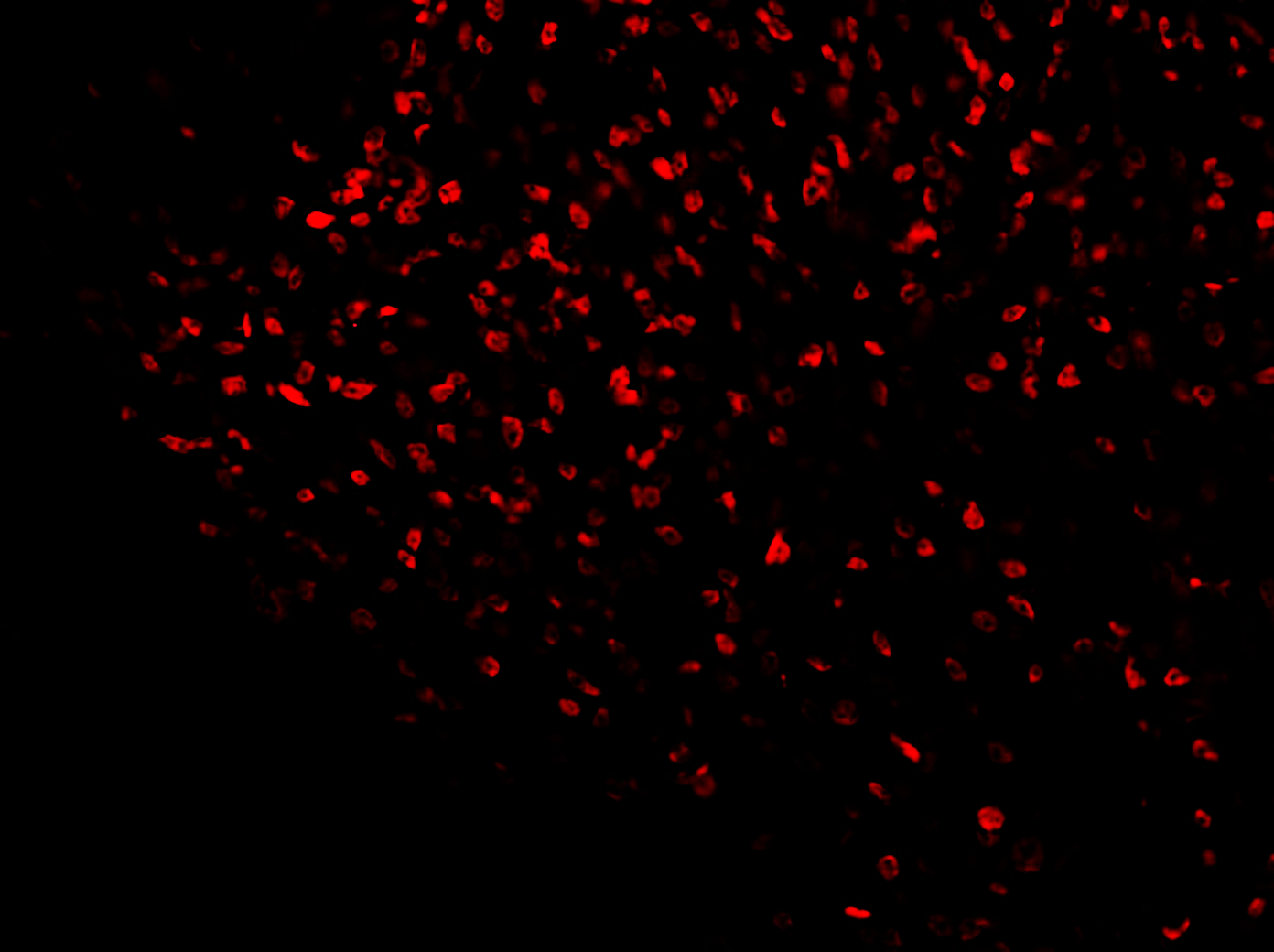


HuR

MERGE


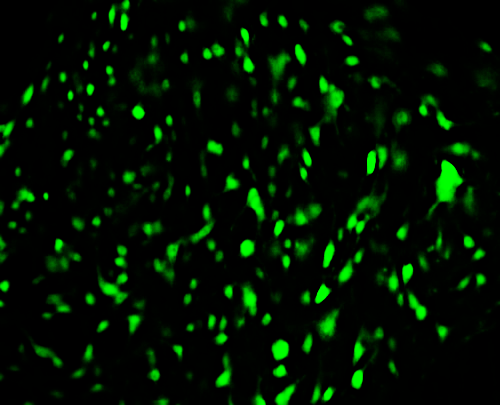

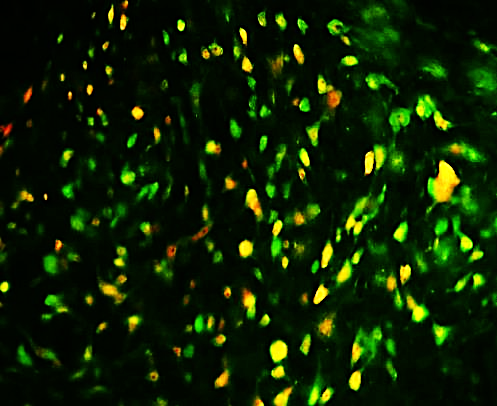


NeuN


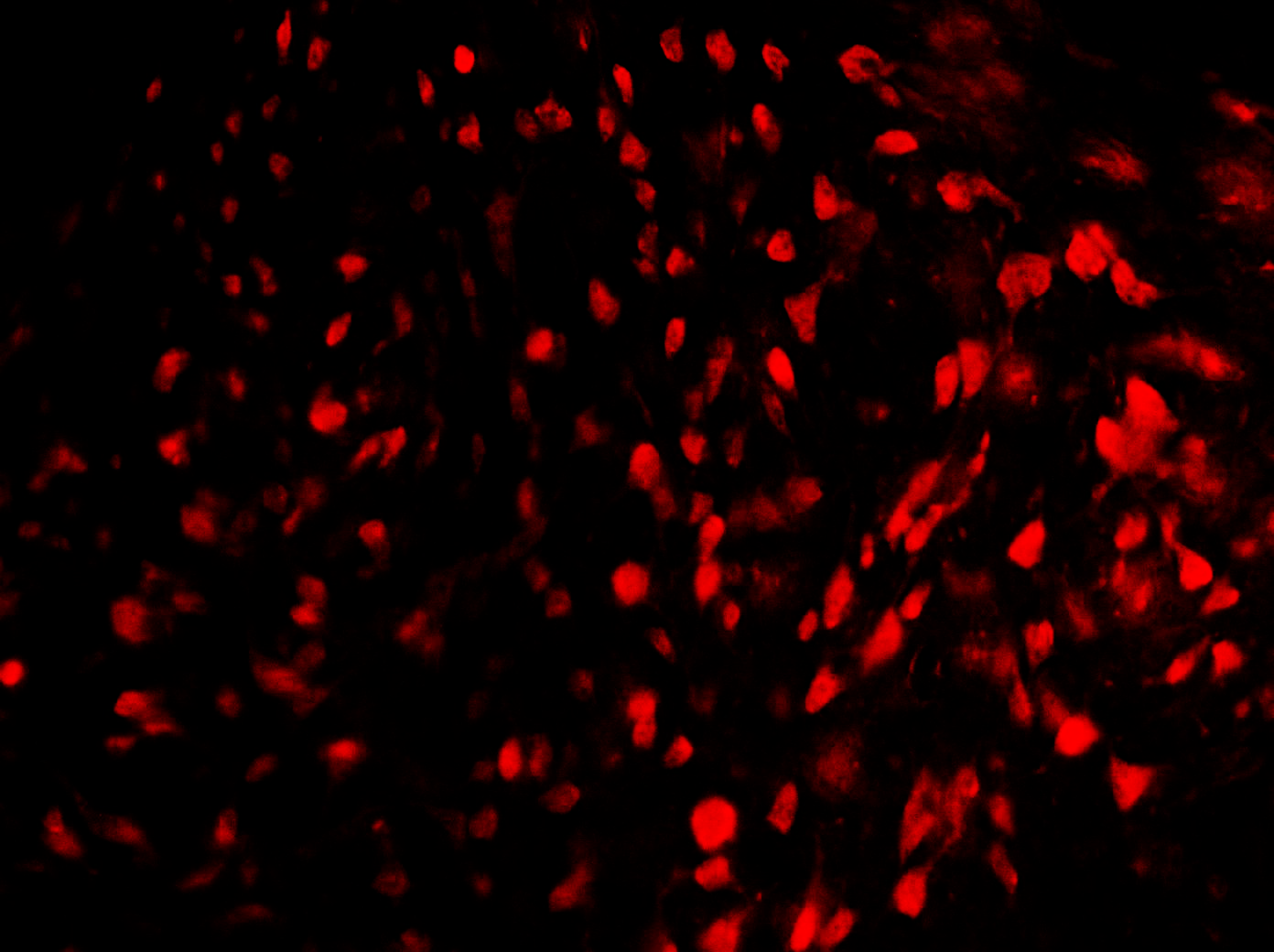


HuR

MERGE

A

B

C

**Supplementary Fig. 4. Cellular localization of HuR and HuD in MOG-EAE spinal cord. (A)** Representative images showing HuD staining in NeuN expressing cell in spinal cord sections. Representative images showing HuR staining in NeuN (B) and CD11b (C) expressing cell in spinal cord sections.
